# Evaluation of CHROMagar™-Serratia agar, a new chromogenic medium for the detection and isolation of *Serratia marcescens*

**DOI:** 10.1007/s10096-021-04328-w

**Published:** 2021-08-07

**Authors:** Blanca Pérez-Viso, Sonia Aracil-Gisbert, Teresa M. Coque, Rosa del Campo, Patricia Ruiz-Garbajosa, Rafael Cantón

**Affiliations:** 1 Servicio de Microbiología, Hospital Universitario Ramón y Cajal and Instituto Ramón y Cajal de Investigación Sanitaria (IRYCIS), Madrid, Spain; 2 Red Española de Investigación en Patología Infecciosa (REIPI), Instituto de Salud Carlos III, Madrid, Spain; 3 Centro de Investigación Biomédica en Red de Epidemiología y Salud Pública (CIBER-ESP), Instituto de Salud Carlos III, Madrid, Spain

**Keywords:** *Serratia marcescens*, Chromogenic medium, Sensitivity and specificity

## Abstract

A comparative analysis of the performance of the new selective chromogenic CHROMagar™-Serratia culture medium for detection and isolation of *Serratia marcescens* was undertaken. A total of 134 clinical isolates (95 *S. marcescens* with and without carbapenemase production and 39 non-*S. marcescens* isolates) and 96 epidemiological samples (46 rectal swabs and 50 from environmental surfaces) were studied. Diagnostic values when compared with CHROMagar™-Orientation medium were 96.8% sensitivity, 100% specificity, 100% positive predictive value and 88.5% negative predictive value. In conclusion, CHROMagar™-Serratia shows an excellent ability for differentiation of *S. marcescens* among clinical isolates and in environmental samples.

**Supplementary Information:**

The online version contains supplementary material available at 10.1007/s10096-021-04328-w.

During the last years, *Serratia marcescens* has become an important nosocomial pathogen and is one of the microorganisms, along with *Klebsiella pneumoniae*, associated with intensive care unit (ICU) outbreaks. It has been particularly recovered in clinical samples from immunosuppressed patients and in those admitted in neonatal ICUs [1–3].

*S. marcescens* is the most frequent species of *Serratia* and causes a wide spectrum of human clinical infections [2]. Although not included in the ESKAPE acronym [4], it is considered a multi-drug resistant (MDR) species within the *Enterobacterales.* Beta-lactamase production represents the most common resistance mechanisms in *S. marcescens,* including AmpC β-lactamase, extended spectrum β-lactamases and carbapenem-hydrolysing enzymes. Moreover, it possesses high rates of acquired resistance to fluoroquinolones and aminoglycosides and is intrinsically resistant to colistin and nitrofurans [5–7].

Within this context, commercial systems for direct detection and identification of *S. marcescens* isolates have been marketed. CHROMagar™-Serratia (CHROMagar Paris, France) is a new selective chromogenic medium for detection and isolation of *S. marcescens*, irrespective of their antimicrobial susceptibility This selective medium is inhibitory for many microorganisms (mostly Gram-positive and yeast), and the interpretation of the growth of colonies is as follows: *S. marcescens* as turquoise to metallic blue, *Pseudomonas* spp*.* as natural pigmentation, and *Morganella morganii* as brown. Other Gram-negatives, including *Proteus* spp. and *Providencia* spp., and Gram-positives as well as yeast are inhibited (https://www.chromagar.com/clinical-microbiology-chromagar-serratia-focus-on-serratia-marcescens-87.html?PHPSESSID=62d014b81cdb556a5ccc551351b2b0ab).

The aim of our study was to evaluate the performance of this medium using a well-characterized collection of isolates and surveillance epidemiological samples prospectively processed (November 2019–January 2020) in the Microbiology Department of Ramón y Cajal University Hospital in Madrid, Spain. We assessed the specificity and sensitivity and determining predictive values to test its efficacy in the clinical setting. Preparation of plates was performed following the manufacturer’s instructions. The ethical committee of our hospital approved the study (ref. 274/19), and all samples were anonymized.

Overall, 134 isolates and 96 samples were used for the evaluation and were seeded directly on the agar plates. The study was divided in two stages: (1) evaluation of the growth of *S. marcescens* and non-S. *marcescens* isolates on the chromogenic medium and (2) evaluation of the growth of *S. marcescens* isolates potentially present in epidemiological surveillance and environmental samples. Workflow is summarized in Fig. 1.

**Fig. 1:**
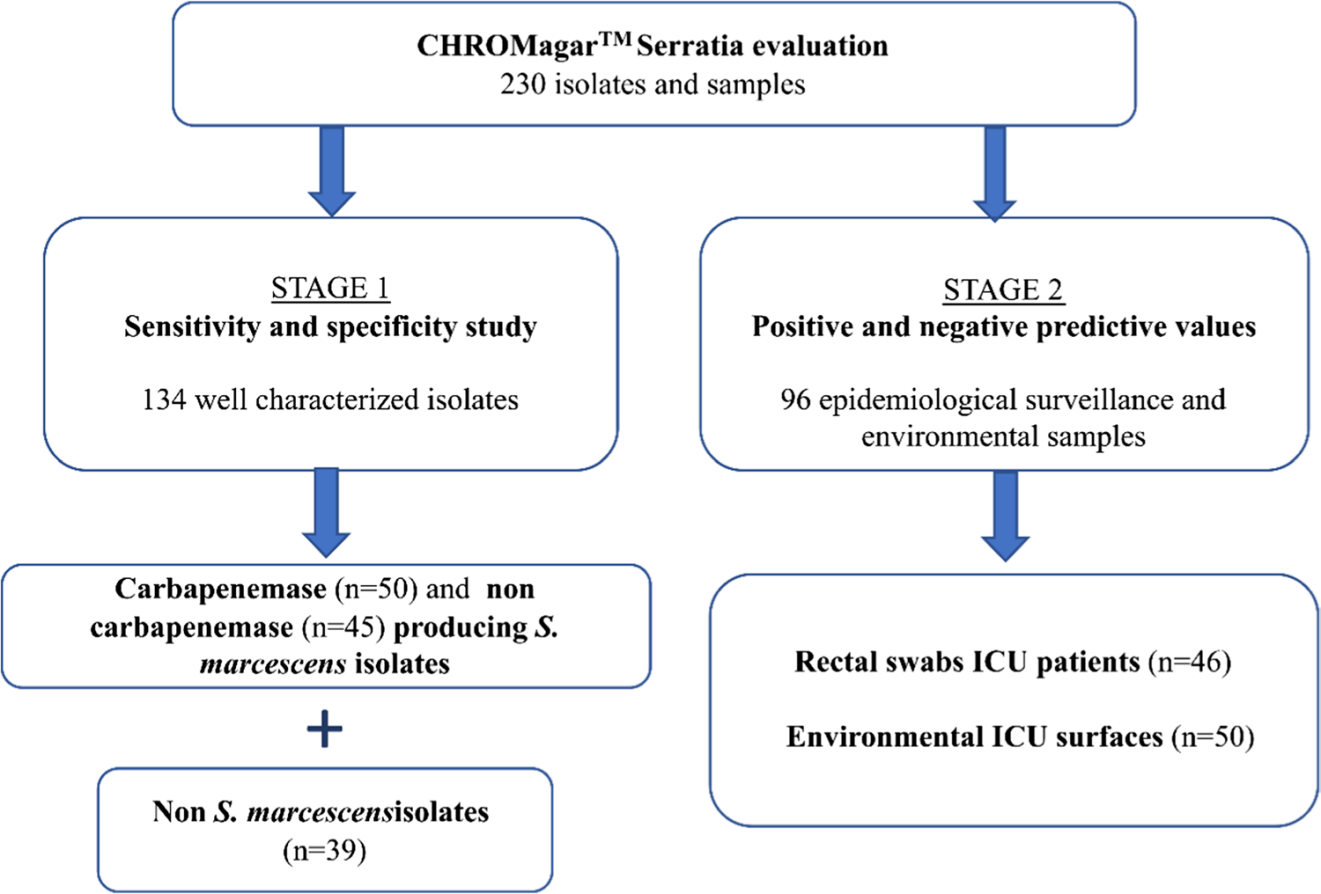
Bacterial isolates and samples used in CHROMagar™-Serratia chromogenic medium evaluation

In stage 1, we determined sensitivity and specificity of the CHROMagar™-Serratia medium using three different sets of isolates: (I) *S. marcescens* isolates obtained from blood cultures (*n* = 45); (II) carbapenemase-producing *S. marcescens* isolates recovered from clinical and surveillance samples (*n* = 50); and (III) a set of isolates (*n* = 39) obtained from blood cultures with an identification different from *S. marcescens*, including Gram-positive (1 *Enterococcus faecium*, 2 *Enterococcus faecalis*, 1 *Enterococcus raffinosus*, 2 *Staphylococcus aureus,* 1 *Staphylococcus hominis*) and Gram-negative (12 *Escherichia coli*, 4 *Klebsiella pneumoniae*, 4 *Morganella morganii*, 1 *Acinetobacter baumannii*, 1 *Acinetobacter pittii*, 2 *Proteus mirabilis*,1 *Moraxella catarrhalis*, 1 *Pseudomonas aeruginosa*, 1 *Enterobacter cloacae*, 2 *Citrobacter freundii*, 1 *Citrobacter koseri*, 1 *Providencia stuartii*, 1 *Stenotrophomonas maltophilia*) microorganisms. Sets I and II of isolates permitted evaluation of the sensitivity of the medium and determination of potential false-negative results; the aim of including carbapenemase-producing and non-carbapenemase-producing *S. marcescens* isolates is to demonstrate if CHROMagar™-Serratia medium is able to detect the presence of *S. marcescens* when harbouring these resistance mechanisms. Set III of isolates allowed to evaluate the specificity of the medium and to determine potential false-positive results.

On the other hand, in stage 2, we determined the detection performance of the CHROMagar™-Serratia medium using samples recovered from environmental abiotic surfaces (sinks and drains) collected in the ICU environment (*n* = 50) and rectal swabs obtained in routine epidemiological surveillance studies from ICU patients with the aim to detect colonization by MDR microorganisms (*n* = 46). These samples allowed the evaluation of positive and negative predictive values.

Besides, all isolates and samples included in our study were seeded in parallel in CHROMagar™-Orientation Medium (BD, USA) which is a general differential medium mainly used in urine specimens. This medium can also partially differentiate *S. marcescens* colonies (turquoise colour) as they are grouped in the same colour grade with *Klebsiella* and *Enterobacter* spp. Plates of this medium were incubated at 37 °C for 24 h.

All colony growths from both stages were submitted for identification confirmation by MALDI-TOF (Bruker Daltonics, Germany) [8]. Moreover, 16S rRNA PCR amplification and sequencing were used for further identification when needed [9].

Overall results of *S. marcescens* growth, comparing both culture media, are presented in Table 1.

**Table 1:** Growth in CHROMagar™ Serratia and CHROMagar™-Orientation

Isolates and samples (*n* = 230)	Growth of *S. marcescens* in:		Growth of no *S. marcescens* in:	
	CHROMagar™-Serratia	CHROMagar™-Orientation	CHROMagar™-Serratia	CHROMagar™-Orientation
*S. marcescens* isolates:				
- Non-carbapenemase-producing (*n* = 45)	42 (93.3%)	42 (93.3%)	0 (0.0%)	0 (0.0%)
- Carbapenemase-producing (*n* = 50)	50 (100%)	50 (100%)	0 (0.0%)	0 (0.0%)
No-*Serratia* isolates:				
- Gram-negatives (*n* = 32)	0 (0.0%)	0 (0.0%)	5 (15.6%)^1^	28 (87.5%)
- Gram-positives (*n* = 7)	0 (0.0%)	0 (0.0%)	0 (0.0%)	6 (85.7%)
Rectal swabs (*n* = 46)	1 (2.2%)^2^	1 (2.2%)^2^	3 (6.52%)^3^	44 (95.6%)
ICU environmental surfaces (*n* = 50)	43 (86%)^4^	43 (86%)	3 (6%)	10 (20%)^5^

^1^4/4 M*. morganii* y 1/1 *P. aeruginosa* grew in CHROMagar™ Serratia but with brown and white colour, respectively, different than turquoise-blue which is the one expected for *S. marcescens* growths; ^2^*S. marcescens* and *P. aeruginosa* grew simultaneously in CHROMagar™-Serratia in one rectal swab. ^3^Two more *P. aeruginosa* grew CHROMagar™-Serratia. ^4^Simultaneous growth of *P. aeruginosa* in 3 samples in which *S. marcescens* also grew. ^5^Additional growth in CHROMagar™-Orientation among others of *E. coli*, *K. pneumoniae*, *E. cloacae*, *C. freundii* and *E. faecium.*

In stage 1 of the work, in which we included 134 isolates (95 *S. marcescens* isolates and 39 non-*S. marcescens* isolates), we detected the presence of *S. marcescens* in 92 isolates, both in CHROMagar™-Serratia and CHROMagar™-Orientation. In the group of non-*S. marcescens*, CHROMagar™-Serratia was able to inhibit the growth of all species except for *M. morganii* and *P. aeruginosa* which were expected to grow but in a different colour (brown and white, respectively) than *S. marcescens* isolates, while in CHROMagar™-Orientation, all species tested were able to grow. The inhibition of most of the microorganisms implies a relevant characteristic of CHROMagar™-Serratia. Nevertheless, we observed negative growths of 3 *S. marcescens* isolates in both chromogenic media. Interestingly, false-positive growths were absent, and the sensitivity and specificity of CHROMagar™-Serratia were 96.9% and 100%, respectively.

In stage 2 in which we included 96 samples (50 environmental surfaces and 46 surveillance rectal swabs), we did not detect the growth of *S. marcescens* in 46 samples in CHROMagar™-Serratia, but we isolated different species in CHROMagar™-Orientation medium. These results confirmed that the new culture medium was able to inhibit other species different from *S. marcescens* when using samples containing complex communities. Nevertheless, in 6 samples, we did not obtain any growth in either culture medium. Taking this into account, positive and negative predictive values were 100% and 88.5%, respectively.

We must underline that in 6 isolates of the non-carbapenemase-producing *S. marcescens* group and in 3 environmental samples where *S. marcescens* was isolated, the colour of the colonies was pink and not turquoise-blue as expected, although the fact of having growth on the plate of CHROMagar™-Serratia already implies the possible presence of *S. marcescens* colonies (Figure S1).

The limit of detection (LOD) of *S. marcescens* in CHROMagar™-Serratia was assessed using 4 isolates, 3 carbapenemase-producing *S. marcescens* isolates, and the ATCC 13,880 (NCTC 10,211) *Serratia marcescens* strain. A suspension in saline NaCl 0.9% in a density of 0.5 McFarland (*ca.* 2 × 10^8^ CFU/ml) was used, followed by serial tenfold dilutions. Limit of detection of the medium was ~ 1 × 10^1^ CFU/ml in the four isolates tested.

Different methods have been used over the years for the identification of *Serratia* species, including conventional biochemical test and API galleries, bacterial typing, and currently MALDI-TOF mass spectrometry. Although chromogenic media have been mainly developed for the isolation of specific bacterial species, they can also be used for bacterial presumptive identification [10]. In this study, we evaluated the CHROMagar™-Serratia chromogenic medium for both purposes. The potential interest of including this medium in the routine of clinical microbiology laboratories is to facilitate the investigation and/or detection of outbreaks caused by MDR *S. marcescens*, particularly in the hospital setting where not only patients are involved but also environmental reservoirs. All *S. marcescens* isolates included in our study were associated with patients in the ICU or with the nosocomial environment. Part of the tested collection were MDR isolates, including carbapenemase producers.

As far as we know, this is the first study testing the performance of CHROMagar™-Serratia in the detection and isolation of *S. marcescens* and its ability for the inhibition of other microorganisms. We must point out that despite the positive results in the evaluation on the performance of CHROMagar™-Serratia, further MALDI-TOF identification was needed to confirm growth colonies. The sensitivity of CHROMagar™-Serratia was close to 97% with 100% of specificity. Note that three false-negative results were obtained with no one false-positive result. Moreover, positive and negative predictive values were 100% and 88.5%, respectively. It should be noted that 100% concordance was observed when compared CHROMagar™-Serratia medium with CHROMagar™-Orientation medium, but the main advantage of the evaluated medium lies on its specific selective properties, facilitating direct detection of *S. marcescens* isolates (colour growth).

On the contrary, we observed 6 *S. marcescens* isolates with pink colony growth both in CHROMagar™-Serratia and CHROMagar™-Orientation media in which natural production of prodigiosin conferring a red pigmentation could be the explanation of the colour of the colonies [11, 12]. A similar observation was obtained with 3 rectal swabs.

In summary, this study evaluating the use of CHROMagar™-Serratia medium shows an excellent ability to differentiate *S. marcescens* clinical isolates allowing us to differentiate between its growth and other Gram-negative ones in an easy way of interpretation. Moreover, this medium was also adequate for the detection of *S. marcescens*, including carbapenemase producers, in epidemiological and environmental samples in the clinical setting. This characteristic gives an advantage to this chromogenic medium for its implementation in clinical microbiology laboratories when *S. marcescens* is involved in nosocomial outbreaks in which both clinical and environmental samples need to be investigated.

## Supplementary Information

Below is the link to the electronic supplementary material.

Supplementary file1 (ODT 1494 KB)
