# Supplementary material for: Evaluation of CHROMagar™-Serratia agar, a new chromogenic medium for the detection and isolation of Serratia marcescens
Source: Eur J Clin Microbiol Infect Dis. 2021 Aug 7;40(12):2593–6. doi: 10.1007/s10096-021-04328-w (PMC8590650; doi:10.1007/s10096-021-04328-w)
Supplement: Supplementary file 1 [file 10096_2021_4328_MOESM1_ESM.odt]

Figure S1: (1) growth of S. marcescens both in CHROMagarTM Orientation (left) and CHROMagarTM Serratia (right) with the turquoise blue colour expected in both culture medium; (2) growth of S. marcescens both in CHROMagarTM Orientation (left) and CHROMagarTM Serratia (right) in one of the three environmental samples in which growth colour was pink; (3) growth of E.coli in CHROMagarTM Orientation (left) and inhibition of its growth in CHROMagarTM Serratia (right).
